# A concern on in-phantom photon energy response of luminescence dosimeters for clinical applications

**DOI:** 10.4103/0971-6203.71756

**Published:** 2010

**Authors:** A. S. Pradhan

**Affiliations:** Editor-in-Chief, JMP C/o AMPI, CT & CRS, BARC, Anushaktinagar, Mumbai - 400 094, India. E-mail: ambika.s.pradhan@gmail.com

Dosimeters based on luminescence properties of solids[1–3](e.g. optically stimulated luminescence dosimeters [OSLDs], radiophoto luminescence dosimeters, plastic scintillator dosimeters, thermoluminescence dosimeters [TLDs] etc.) are finding wider application in clinical dosimetery. This is attributed to the availability of these dosimeters in very small sizes to provide high spatial resolution and their capability of covering a very wide dose range and with high precision (< 1 %). These dosimeters are highly suited for in vivo and in-phantom dosimetry for placing in the vicinity of the organ being treated, where the dose decreases rapidly with distance from a source or from target/treatment volume. In the context of the photon (X- and gamma rays) energy response correction factors for luminescence dosimeters, a recent publication[4] demonstrated and concluded that Monte Carlo (MC) calculations/simulations[5] for the most widely used LiF TLD do not accurately predict the relative response as a function of photon energy. The differences (up to 10%) in measured and MC-calculated TLD responses of LiF TLD are because the MC simulations do not properly account for the solid state physics of the luminescence mechanism. The ratio of measured TLD light output per unit air kerma as a function of photon energy to the MC-calculated TLD dose per unit air kerma indicates that the light output of LiF TLD-100 (LiF:Mg, Ti) is not directly proportional to the dose of TLD over the range of photon energies from 10 keV to 1,000 keV. The study[4] also highlighted the inadequacy of the usual practice of using the reference irradiation taken from a higher energy source such as ^60^Co or a 4 or 6 MV linear accelerator (which have well-established dosimetry protocols based on absorbed dose to water) for the use of TLDs for various dosimetric purposes, including dosimetry of sources used in brachytherapy (BT). A recent intercomparison of solid state dosimeters demonstrated[6] that the depth-dependent change in the sensitivity of LiF TLD chips due to the change in the photon energy spectra is one of the reasons for the uncertainty in the measurements of doses. It is timely to look into the aspects of in-phantom photon energy dependence correction factors of luminescence dosimeters.

Unlike ionization chambers and other dosimeters wherein ion pairs produced by radiation are directly measured to arrive at the dose, in the case of dosimeters based on luminescence properties of solids, only the light signal caused by the ionization is measured and related to the quantity of the radiation. The emission of light is a result of radiative recombination of holes and electrons at the luminescent centers in the dosimetry material. The luminescence efficiency is influenced by a number of parameters e.g., trapping of holes and electrons at the traps and other defect centers along the track of secondary electrond produced by radiation, their interaction during `release and recombinations at the luminescence center. The ionizing density of radiation (which influences the spatial distribution of traps and other defect complexes for the radiative recombination, the microscopic dose distribution along the track and other processes) changes with radiation energy which keeps changing with the passage of radiation in a phantom. When the luminescence efficiency of a dosimeter changes with the change in the quality/energy of the radiation in the phantom, the photon energy dependnce correction factors arrived on the basis of the ratio of mass energy absorption coefficients is altered. For theoretical calculations of these corrections factors, there is no universal way to crrectly speculate the change in the luminescence efficiency from the physical or chemical properties of the dosimeter material. Even for the same material, the photon energy response could be significantly altered by a small change in the concentration of the dopants (ppm level) which are responsible for the luminescence properties. The most exciting example is the change in the photon energy response (light per unit absorbed dose) of LiF TLDs when doped (in trace quantities) with Mg and Ti in one case and with Mg, Cu and P in the other case. LiF:Mg, Ti exhibits[4,7] an over-response of about 10% (more than the predicted response calculated based on the ratio of mass energy absorption coefficients) for photons below 250 keV as compared to its response to 1,250 keV photons (^60^Co gamma rays), whereas LiF:Mg, Cu, P exhibits an under-response up to 30%.[7] Even for 670 keV photons (^137^Cs gamma rays), LiF:Mg, Ti exhibits an over-response and LiF:Mg, Cu, P exhibits an under-response as compared to their responses to 1,250 keV photons (^60^Co gamma rays).[7] This effect of change in the energy response is not limited to LiF TLDs but has been observed in other luminescence dosimeters[8] also. Apart from LiF:Mg, Cu, P, examples of significantly reduced responses (as compared with the predicted responses) are those of dosimeters based on plastic scintillators[8] and the 150°C glow peak of CaF_2_:Tm TLD,[9] whereas examples of over-responses are also equally significant, such as the response of the 240°C glow peaks of LiF:Mg, Ti TLD[10] and CaF_2_:Tm TLD.[9]

For the use of LiF:Mg, Ti in dosimetry of ^192^Ir high-dose rate (HDR) afterloading sources, Meigooni et al.[11] appear to be the first to point out that LiF:Mg, Ti exhibits an over-response that varies with the depth in the phantom due to the shift of the photon spectrum to lower energies with increasing depths in the phantom material. This over-response from the change in the spectra at different depths (due to multiple scattering) was estimated to be as much as 8.5% at a depth of 10 cm as compared to a value at 1 cm depth. These depth-dependent correction factors were accepted in some cases to correct for the radial dose measurements, but several other authors did not use these correction factors and, at one point of time in the past, this became a topic of debate.[12,13] To explain this discrepancy, another study[14] was carried out in which LiF TLDs were placed at different depths in the phantom at points for which the measured values of absorbed doses were separately and simultaneously determined by using a 0.3 cm^3^ ion chamber. The over-response of LiF TLD-100 rods was thus found to be not exceeding 2.5% at the depth of 10 cm in the phantom as compared to the depth at 1 cm[14].

This clearly demonstrates that for all luminescence dosimeters, photon energy dependence correction factors need to be experimentally determined and the calculated values based on the ratio of mass energy absorption coefficients should not be used. In general, the experimental measurements of photon energy responses by most authors has remained limited to exposures free in air (by holding the dosimeters in appropriate build-up materials) in defined beams of X- and gamma ray energies and using air-Kerma values (free in air). To minimize the discrepancies, it is advisable to determine these correction factors by placing the dosimeters in the phantom at the points of interest to simulate the actual situations as closely as possible. Evidently, this topic has now become of significant concern and newer studies[4,7] are impressing on the experimental determination of in-phantom depth-dependent photon energy response correction factors, failing which unacceptable inaccuracies might creep in.
